# Predicting drug–drug interactions between ayahuasca alkaloids and SSRIs using physiologically based pharmacokinetic modeling

**DOI:** 10.3389/fmolb.2026.1768402

**Published:** 2026-02-18

**Authors:** Gabriella de Souza Gomes Ribeiro, Beatriz Aparecida Passos Bismara Paranhos, Fabiane Dörr, Maurício Yonamine, Bianca Villanova, Lorena Terene Lopes Guerra, Adrieli Oliveira Raminelli, Jose Augusto Silva Reis, Caio Cesar de Paula, Anna Beatriz Vicentini Zacharias, Jaime Eduardo Cecílio Hallak, Rafael Guimarães dos Santos, Frederico Severino Martins, Tania Marcourakis

**Affiliations:** 1 Department of Clinical and Toxicological Analyses, University of Sao Paulo, Sao Paulo, Brazil; 2 Department of Neurosciences and Behavioral Sciences, Ribeirao Preto Medical School, University of São Paulo, Ribeirao Preto, Brazil; 3 National Institute of Science and Technology - Translational in Medicine (INCT-TM), CNPq, Ribeirao Preto, Brazil

**Keywords:** ayahuasca, DDI, harmine, N,N-Dimethyltryptamine, PBPK modeling, SSRI

## Abstract

**Introduction:**

Ayahuasca is a psychedelic preparation containing N,N-dimethyltryptamine (DMT) and the β-carboline harmine (HRM), a reversible monoamine oxidase A inhibitor that enables DMT oral bioavailability. The increasing concomitant use of ayahuasca with selective serotonin reuptake inhibitors (SSRIs) has raised concerns about potential pharmacokinetic and pharmacodynamic interactions, particularly because fluoxetine and paroxetine are strong CYP2D6 inhibitors and DMT and HRM undergo CYP-mediated metabolism. This study aimed to develop and validate physiologically based pharmacokinetic (PBPK) models to predict the impact of SSRI coadministration on the systemic exposure of DMT and HRM.

**Methods:**

PBPK models for DMT and HRM were developed and qualified using plasma concentration–time data from a controlled clinical study in which six volunteers received oral ayahuasca. Models for fluoxetine, norfluoxetine, and paroxetine were developed based on published clinical data and incorporated enzyme inhibition parameters to represent their inhibitory potential. Drug–drug interaction simulations were performed under acute and chronic SSRI dosing conditions.

**Results:**

Both fluoxetine and paroxetine increased HRM exposure and produced moderate increases in DMT systemic concentrations. These effects were consistent with CYP2D6 inhibition and enhanced monoamine oxidase A blockade. The simulations demonstrated that SSRI coadministration alters the pharmacokinetic profiles of ayahuasca alkaloids under both acute and chronic dosing scenarios.

**Discussion:**

The findings suggest a clinically relevant interaction between ayahuasca and SSRIs, as even modest increases in DMT exposure may intensify serotonergic effects in individuals receiving antidepressant therapy. This study provides a mechanistic and quantitative framework for assessing interaction risks between ayahuasca alkaloids and SSRIs, supporting clinical decision-making and harm-reduction strategies in contexts where controlled drug–drug interaction studies are not feasible.

## 1 Introduction

Drug–drug interactions (DDIs) between synthetic medicine are well known and extensively explored in both preclinical and clinical contexts. However, interactions between natural products and synthetic medicaments remain largely outside the radar of clinical investigations, despite the increasing concomitant use of herbal medicines, supplements, and functional foods with other pharmacotherapies. Such combinations may alter drug pharmacokinetics or pharmacodynamics, potentially compromising thesafety and efficacy (Maldonado and Lemasters, 2012). This issue is particularly relevant in antidepressant therapy, where patients may self-administer natural products together with prescribed medications without informing their physicians. Many of medicaments are cytochrome P450 (CYP)-mediated metabolism, and complexity is further amplified when natural products modulate metabolic pathways such as CYP2D6, amplifying the DDI of drugs like fluoxetine (FL) and paroxetine (PR) (Zhang et al., 2023).

**FIGURE 1:**
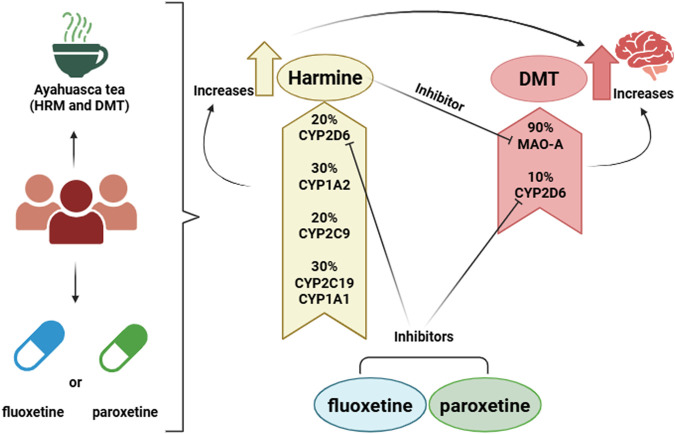
Schematic representation of the metabolism of DMT and HRM present in ayahuasca and their potential interactions with fluoxetine (FL) and paroxetine (PR). HRM inhibits MAO-A (black arrow), increasing brain DMT concentrations. The percentages indicate the relative contribution of each cytochrome P450 isoenzyme to the metabolism of HRM and DMT. Since FL and PR strongly inhibit CYP2D6, they may raise HRM levels and, indirectly, enhance DMT exposure.

Within this context, ayahuasca represents a clinically relevant and increasingly prevalent case. It is a psychedelic beverage traditionally used by South American indigenous groups in rituals and therapy (McKenna and Riba, 2015). He brew is typically prepared by prolonged decoction of the vine *Banisteriopsis caapi* (B. caapi), which contains the β-carbolines harmine (HRM), tetrahydroharmine (THH), and harmaline (HML)—reversible inhibitors of monoamine oxidase A (MAO-A)—together with the leaves of *P. viridis* (*Psychotria viridis*), a source of the hallucinogen N,N-dimethyltryptamine (DMT), a serotonin receptor agonist (McKenna and Riba, 2015). Orally administered DMT is rapidly degraded by MAO-A, resulting in negligible bioavailability. However, concomitant ingestion of β-carbolines inhibits MAO-A, enabling the absorption of DMT and its penetration into the central nervous system. HRM undergoes O-demethylation primarily via CYP1A1, CYP1A2, CYP2D6, CYP2C9, and CYP2C1 (Hamill et al., 2019), while DMT itself also undergoes secondary metabolism via CYP2D6 (McIlhenny et al., 2012). The β-carboline HRM is considered the primary determinant of functional MAO-A inhibition due to its inhibitory potency and the plasma concentrations achieved (Herraiz et al., 2010). HML also exhibits inhibitory activity toward MAO-A; however, its systemic exposure is significantly lower compared with HRM, with plasma concentrations approximately fivefold lower. This limits its relative contribution to overall MAO-A inhibition (Riba et al., 2003). THH, although detectable in plasma following ayahuasca ingestion, also exhibits very low inhibitory activity toward MAO-A compared with HRM and is therefore considered of minimal relevance for the functional inhibition of this enzyme (Riba et al., 2003). Accordingly, the present study focused on HRM as the principal modulator of MAO-A activity. Fluoxetine, a selective serotonin reuptake inhibitor (SSRI) available for more than 40 years, undergoes hepatic metabolism via CYPs, generating the active metabolite norfluoxetine (NFL) (Dorado et al., 2004). Paroxetine, another SSRI of the phenylpiperidine class, is extensively metabolized by CYP2D6 and CYP3A4 and is itself a potent CYP2D6 inhibitor (Bloomer et al., 1992). Given these shared metabolic pathways, the concomitant use of ayahuasca and SSRIs raises important concerns regarding both pharmacokinetic and pharmacodynamic interactions. This process is complex, as HRM acts as an MAO-A inhibitor and is also a substrate of CYP2D6. Thus, coadministration with strong CYP2D6 inhibitors such as FL and PR may increase systemic HRM exposure and, indirectly, DMT exposure through enhanced MAO-A inhibition. Furthermore, individuals with depressive disorders undergoing treatment with synthetic antidepressants may seek alternative or natural therapies, such as the ritualistic or therapeutic use of ayahuasca. In many cases, these patients may not inform their physicians about the concomitant use of the brew, which increases the risk of severe interactions, including serotonin syndrome. Figure 1 illustrates these interactions.

Advances in modeling and simulation techniques have provided a more detailed and quantitative understanding of pharmacokinetic processes, supporting more informed decision-making in the assessment of DDI risks. Physiologically based pharmacokinetic (PBPK) models employ a mechanistic approach that integrates the physicochemical properties of drugs with established knowledge of human physiology, allowing a quantitative representation of their absorption, distribution, metabolism, and excretion (ADME) (Martins et al., 2023). This approach enables a mechanistic investigation of potential drug interactions, distinguishing, for example, the effects caused by SSRI inhibition on the ADME pathways of DMT and HRM. Moreover, PBPK models serve as a valuable tool for optimizing the design and conduct of clinical studies in which drug interactions may be present (Schaller et al., 2025). This modeling strategy can support the design of future clinical DDI studies, guide dose adjustment strategies, and, in certain situations, even replace unnecessary clinical trials, thus contributing to safer and more efficient drug development pipelines (Choi et al., 2025; Yang et al., 2025).

The objective of this work was to apply PBPK modeling to explore and predict potential drug–drug interactions (DDIs) between ayahuasca constituents and the SSRIs fluoxetine and paroxetine. In addition, the DMT and HRM models were previously developed and validated using data from an internal clinical study, providing greater robustness to the predictions. By mechanistically integrating metabolic pathways and enzyme inhibition profiles, this study aims to offer insights into interaction risks that remain insufficiently characterized in clinical settings, supporting safer co-use assessments and guiding the design of future DDI studies.

## 2 Methods

### 2.1 In-house clinical study of DMT and HRM

The study included 6 volunteers (4 men and 2 women), of whom 4 had previous experience with ayahuasca tea. Exclusion criteria included the use of any medication that could interfere with the pharmacokinetics of the compounds present in ayahuasca. The participants had a mean age of 32 ± 11 years, a mean weight of 75 ± 11 kg, and a mean height of 1.71 ± 0.06 m. The ayahuasca used in the study was donated by the religious institution Centro de Regeneração Espiritual Casa de Jesus e Lar de Frei Manuel to the Department of Neurosciences and Behavioral Sciences at Ribeirao Preto Medical School of the University of Sao Paulo (FMRP-USP), where the clinical study was conducted. All volunteers were fully informed about the composition and characteristics of the tea, as well as the potential risks associated with participation. The study was approved by the Research Ethics Committee (CEP) of the Hospital das Clinicas, School of Medicine of Ribeirao Preto, University of São Paulo (Clinics Hospital at FMRP-USP) on 5 February 2024 (CAAE: 69291023.5.0000.5440). All participants signed a written informed consent form. The volunteers received an oral dose of 1 mL/kg of ayahuasca.

Blood samples of 5 mL were collected at the following time points: pre-dose, 20, 40, 60, 90, 120, 180, and 240 min after administration of ayahuasca. Samples were transferred to 4 mL EDTA tubes and immediately centrifuged at 2,000 rpm for 10 min at 4 °C; plasma was separated and stored at −20 °C, then transferred to −80 °C until the day of analysis. The experimental session lasted 280 min, at which point the effects of ayahuasca had subsided. The concentrations of DMT and HRM was were quantified by High-Performance Liquid Chromatography coupled with tandem Mass Spectrometry (LC-MS/MS) according to the guidelines described in Souza et al. (2019). The chemical composition of the administered ayahuasca batch was as follows: 0.67 mg/mL DMT and 1.77 mg/mL HRM.

#### 2.1.1 Quantification of DMT and HRM in plasma

A mixture of 50 µL of human plasma and methanol (1:2) was added to 200 µL of the internal standard DMT-d_6_ (Cerilliant, Round Rock, TX, United States) prepared in methanol as the precipitating solvent; the DMT analytical standard was obtained from Toronto Research Chemicals–TRC (Toronto, ON, Canada). The mixture was vortexed for 10 s and centrifuged at 14,000 rpm for 10 min. Finally, 150 μL of the supernatant was collected, and 5 μL of this solution was injected into the equipment. The chromatographic system (Quattro Premier XE/Acquity UPLC System - Waters), was composed of a C18 column (2.1 mm × 100 mm, 1.7 μm) from Waters Corporation (Milford, Massachusetts, United States). For the chromatographic system, a gradient elution was applied with 1 mM ammonium formate buffer containing 0.1% formic acid (mobile phase A) and 0.1% formic acid in methanol (mobile phase B). The flow rate was maintained at 0.3 mL/min, and the column temperature was set at 40 °C. The elution gradient was as follows: 0–0.5 min, 10% B; 0.5–7 min, 40% B; 7.1–8.0 min, 80% B; 8.1–9.0 min, 10% B; and 9.1–12.0 min, 10% B. The total run time was 12.0 min. The mass spectrometer was operated in Multiple Reaction Monitoring (MRM) mode, considering three transitions for each analyte. MS parameters were set as follows: desolvation gas flow rate, 750 L/h; cone voltage, 40 V; desolvation temperature, 350 °C; source temperature, 120 °C; and capillary voltage, 3.00 V. For the linearity assay, blank human plasma samples were spiked with analytical standards and subsequently extracted. Quantitative analyses of DMT and HRM were performed using weighted linear regression curves constructed from certified standards (DMT from TRC, Toronto Research Chemicals, Toronto, Canada; harmine and related β-carbolines from Cayman Chemical, Ann Arbor, MI, United States) with predefined concentrations. The method followed established criteria for analytical validation, including assessment of the coefficient of determination (*R* ^2^), precision, accuracy, and relative error (RE%) at different concentrations. A linearity curve was constructed encompassing points from 0.1 ng/mL to 350 ng/mL, as published pharmacokinetic data indicate the lowest and highest plasma concentrations of these alkaloids. After the analysis, a pharmacokinetic curve was constructed considering the mean plasma concentration of the volunteers. The data was processed using Microsoft Excel® 2024.

### 2.2 PBPK modeling development and verification

The PBPK simulations were performed using PK-Sim (Open Systems Pharmacology Suite, version 11.3; www.open-systems-pharmacology.org). Physicochemical and biochemical properties of each compound—including molecular weight, pKa, logP, solubility, and plasma protein binding—were retrieved from published sources such as DrugBank, PubChem, and ChEMBL.

Step 1 — Initial model development using published data Structural PBPK models for FL, NFL, and PR were first developed using published clinical pharmacokinetic (PK) data as the primary source for parameterization (Supplementary Tables S5, S6). These datasets were used to refine absorption, distribution, metabolism, and excretion (ADME) parameters and to ensure that the simulated concentration–time profiles adequately reflected the central tendency of observed data. Fluoxetine and NFL models were based on Jeong et al. (2021), whereas the PR model followed the structure described by (Cho et al., 2024). Both studies provided inhibition constants (Ki) for FL, NFL, and PR toward major CYP enzymes. These Ki values were incorporated to mechanistically represent SSRI inhibitory potential on DMT and HRM metabolism. A complete list of all parameters used in the models is provided in Supplementary Tables S2, S3 of the Supplementary Material.

Step 2 — Validation of DMT and HRM models using in-house clinical data Model validation for DMT and HRM was performed using the clinical data from the in-house study conducted at the Ribeirao Preto Medical School of the University of Sao Paulo (FMRP-USP). This dataset, which includes subject demographics, dosing information, and sampling schedule, allowed direct comparison between simulated and observed plasma concentration–time profiles under strictly controlled experimental conditions. Validation using this well-characterized volunteer population ensured greater precision and reduced variability. Details are provided in Supplementary Table S4 (Supplementary Material).

Step 3 — Validation of SSRI models using external clinical datasets For FL, NFL, and PR, external clinical concentration–time profiles were extracted from the literature using WebPlotDigitizer (https://automeris.io/WebPlotDigitizer/). These datasets, detailed in Supplementary Tables S5, S6 (Supplementary Material), were used for independent model qualification. Comparing simulated and observed curves across multiple studies strengthened the robustness and generalizability of the PBPK predictions.

Step 4 — Simulation in virtual populations Following model validation, simulations for FL, NFL, and PR were performed using a virtual population of 100 individuals aged 20–59 years and weighing 60–80 kg. This step incorporated interindividual variability in physiological and metabolic parameters, improving the model’s translational relevance for predicting real-world population responses.

Step 5 — Model qualification and performance criteria Model qualification was based on comparison between predicted and observed pharmacokinetic parameters, specifically the area under the plasma concentration–time curve (AUC_0_–t) and maximum plasma concentration (Cmax). Predictive accuracy was assessed using the mean fold error (MFE), calculated as:

$$\text{MFE}=\frac{\text{Predicted}\;\text{parameter}}{\text{Observed}\;\text{parameter}}$$

MFE values within the commonly accepted range of 0.5–2.0 were considered indicative of adequate model performance.

**FIGURE 2:**
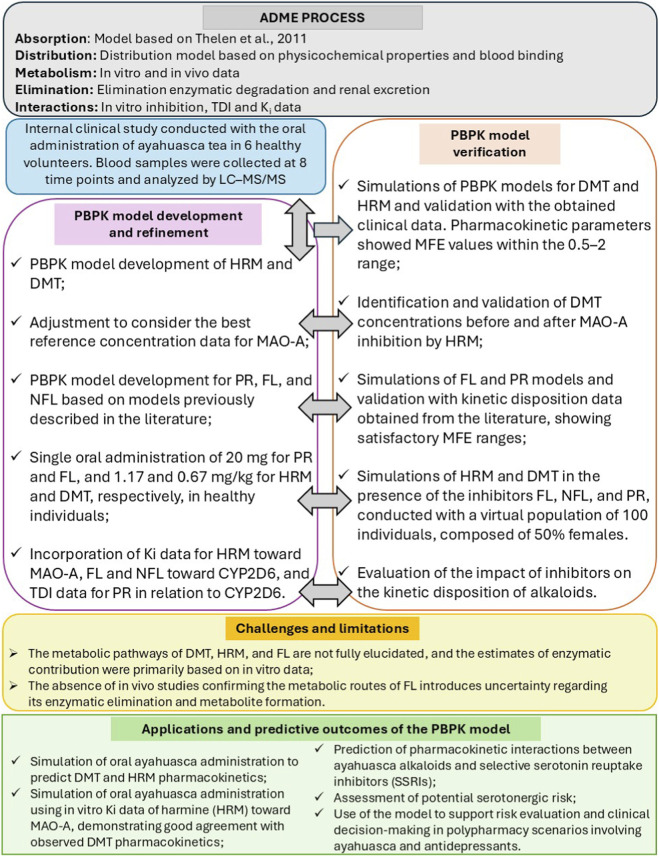
Flowchart illustrating the development, validation, and predictive applications of the PBPK models for ayahuasca alkaloids and SSRIs. The diagram summarizes the steps of the ADME process, the internal clinical study, the construction, refinement, and validation of the models using our clinical study and published data, as well as the main limitations, applications, and clinical implications identified. Figure adapted from Loisios-Konstantinidis et al. (2025).

Step 6 — Overview of modeling workflow The overall strategy for PBPK model development, verification, and application in drug–drug interaction (DDI) simulations is summarized in Figure 2.

### 2.3 Modeling strategy for interaction analysis

For the simulation of enzymatic interaction scenarios, several mechanistic considerations were incorporated to justify the anticipated changes in exposure. First, for HRM, the MAO-A inhibition parameters used in the model were deemed appropriate because the clinical plasma concentration–time data implicitly reflect MAO-A inhibitory effects. This alignment indicates that the available *in vitro* data were suitable for representing HRM’s MAO-A inhibition within the PBPK framework.

Second, for paroxetine (PR), inhibition of CYP2D6 was modeled by considering the relative contribution of this enzyme to HRM metabolism. The observed increase in HRM AUC in the presence of PR was mechanistically supported by PR’s potent CYP2D6 inhibitory effect, consistent with its characterization as a mechanism-based inactivator. *In vitro* Ki and Kinact data were incorporated to reproduce this behavior.

Third, the potential effects of FL and its metabolite NFL on HRM metabolism were evaluated using previously published *in vitro* inhibition parameters. Both compounds act simultaneously as substrates and inhibitors of CYP2D6, resulting in concentration-dependent auto-inhibition and accumulation under repeated dosing regimens. Within the PBPK framework, this behavior emerges naturally from the interaction between CYP2D6-mediated clearance and the incorporated inhibition parameters. However, because Ki values derived from *in vitro* studies may underestimate the effective *in vivo* inhibitory potential of FL and NFL under steady-state conditions, clinically relevant dosing regimens were simulated to capture their sustained inhibitory effect on CYP2D6 activity. Overall, these modeling decisions ensured that the PBPK simulations captured both the immediate and cumulative effects of SSRI-mediated enzyme inhibition on the disposition of HRM and DMT.

### 2.4 Definition of the dosing protocol for inhibitors and substrates used in the DDI predictions

Paroxetine is a time-dependent CYP2D6 inhibitor (mechanism-based inactivator) (Cho et al., 2024). Therefore, the magnitude of its inhibitory effect depends on both the duration of exposure and the cumulative inactivation of the enzyme. For this reason, two PR protocols were simulated: An acute protocol (5 days) to assess early inhibitory effects, and A prolonged protocol (14 days) to evaluate the cumulative inhibition and identify the inhibition plateau. Fluoxetine (FL) was modeled as a reversible inhibitor; however, its active metabolite NFL exhibits a long half-life and contributes substantially to sustained CYP2D6 inhibition (Sagahón-Azúa et al., 2021). Thus, extended simulations were also necessary for FL to capture metabolite accumulation and its impact on substrate exposure.

Time-dependent inhibition for PR was implemented using published Kinact/Ki parameters, enabling mechanistic representation of irreversible enzyme inactivation. Additionally, it is important to consider that individuals seeking ayahuasca may already be undergoing chronic treatment with antidepressants. For this reason, dosing regimens for inhibitors were designed to reflect steady-state or near–steady-state conditions frequently observed in clinical use.

The dosing protocols implemented in the PBPK simulations for each inhibitor and substrate are summarized in Table 1.

**TABLE 1:** Simulated dosing regimens of inhibitors and substrates for DDI evaluation.

Inhibitor (clinical dose)	Substract (clinical trial dose)	Simulated inhibitor regimen	Substrate dosing	Inhibition model
Paroxetine (20 mg QD)	HRM: 1.77 mg/kg DMT: 0.67 mg/kg	20 mg QD Days 1–5 and 1–14	Day 5 and day 14	Mechanism-based inactivation
Fluoxetine (20 mg QD)	HRM: 1.77 mg/kg DMT: 0.67 mg/kg	20 mg QD Days 1–5 and 1–14	Day 5 and day 14	Competitive inhibition

QD, once daily; SD, single dose.

### 2.5 Sensitivity analysis

A sensitivity analysis was performed in PK-Sim® to evaluate the relative impact of key mechanistic parameters involved in MAO-A and CYP2D6 inhibition on the simulated DDI outcomes. Parameter perturbations were conducted using the built-in variation range approach, with a variation range of 0.5 applied to each parameter individually, corresponding to a ±50% change around the reference value.

## 3 Results

### 3.1 Quantification of plasma DMT and HRM

Quantitative analyses of DMT and HRM demonstrated that the weighted linear regression calibration curves exhibited coefficients of determination (*R* ^2^) within the acceptance criteria established for bioanalytical validation. The chromatographic peak areas showed good concordance with the expected concentrations, with relative errors (RE%) remaining below the permissible limits. Representative chromatograms, obtained after raw data processing using MassLynx™ Software V4.1 (Waters Corporation, Milford, MA), are provided in the Supplementary Material (SI).

**FIGURE 3:**
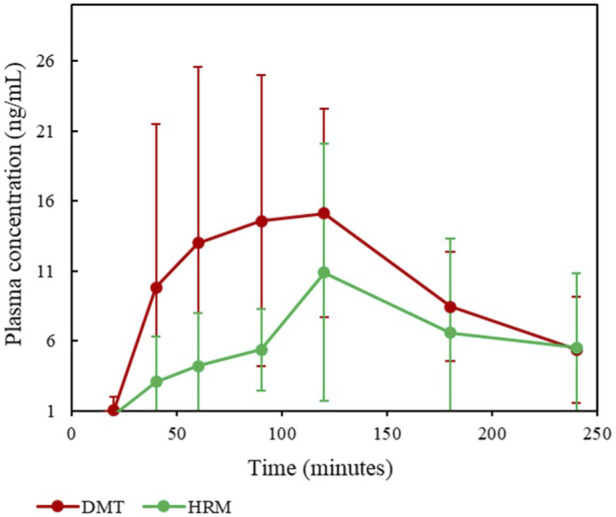
Plasma concentration-time profiles of DMT and HRM following oral administration of ayahuasca in six healthy volunteers. Data are shown as mean ± SEM.

All analytical data were processed using MassLynx™ Software V4.1, and linearity assessments were performed in Microsoft Excel® 2019. The resulting mean plasma concentration–time profiles from the six volunteers are presented in Figure 3.

**FIGURE 4:**
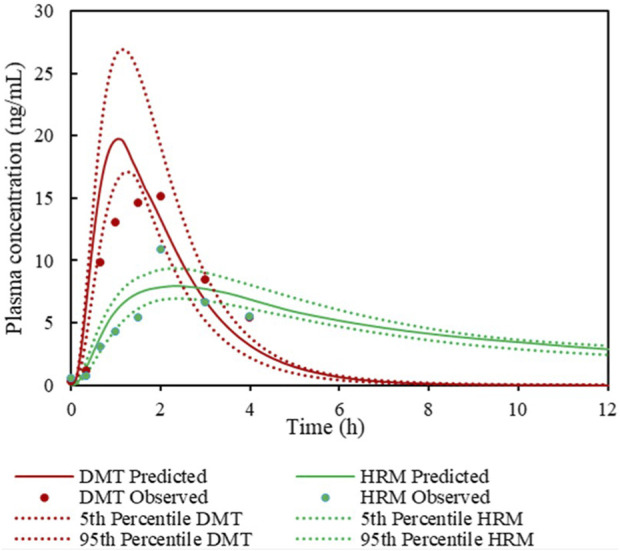
Simulation of plasma concentration–time profiles of DMT and HRM.

Based on these profiles, DMT exhibited an AUC of 38.6 ng h/mL and a Cmax of 15.1 ng/mL. For HRM, the AUC was 23.3 ng h/mL, with a Cmax of 10.9 ng/mL. The variability observed in the plasma concentrations of DMT and HRM shown in Figure 4 may be partially attributed to the limited sample size and interindividual variability. Physiological differences among individuals, such as potential variations in intestinal MAO-A expression, as well as differences in compound absorption and bioavailability, may contribute to the dispersion of the observed data.

### 3.2 Validation of the DMT and HRM models

Both PBPK models demonstrated good predictive performance in describing the pharmacokinetic disposition of DMT and HRM following oral administration of ayahuasca in healthy volunteers. The simulated plasma concentration–time profiles closely reproduced the observed data obtained from the internally conducted clinical study, as illustrated in Figure 4.

For DMT, the mean fold error (MFE) was 0.87 for AUC and 1.3 for Cmax. For HRM, the corresponding MFE values were 1.54 for AUC and 0.72 for Cmax. All values fell within the commonly accepted qualification range (0.5–2.0), indicating that the models reliably captured the key pharmacokinetic characteristics of both alkaloids (Table 2).

**TABLE 2:** Summary of clinical study data used for DMT and HRM. The table presents predicted and observed data, as well as the MFE for the evaluated kinetic parameters. Observed AUC values were extrapolated up to 12 h.

Compound/route of administration	Predicted AUC (ng·h/mL)	Observed AUC (ng·h/mL)	Predicted Cmax (ng/mL)	Observed Cmax (ng/mL)	MFE AUC	MFE Cmax
DMT/Oral dose	42.79	49.16	19.79	14.1	0.87	1.3
HRM/Oral dose	59.51	38.49	7.92	10.9	1.54	0.72

### 3.3 Fluoxetine and paroxetine

**FIGURE 5:**
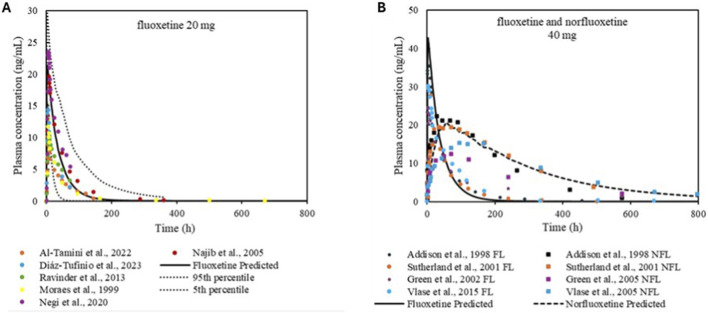
Simulation of plasma concentration–time profiles of FL and NFL. Panel **(A)** shows the simulation of 20 mg of fluoxetine. Panel **(B)** shows the simulation of 40 mg of FL, including the plasma concentrations of both FL and NFL.

The plasma concentration–time profiles following oral administration of FL are shown in Figure 5. The mean Cmax for FL and NFL were 1.4 and 1.2, respectively. Calculated MFE for FL was 1 and for NFL was 1.1, both within the recommended range (Table 3).

**TABLE 3:** Summary of clinical study data used for FL and NFL. The table reports predicted and observed data, along with the MFE for the evaluated kinetic parameters. Observed AUC values were extrapolated up to 800 h.

Compound/route of administration	Dose	Ref	Predicted AUC (ng·h/mL)	Observed AUC (ng·h/mL)	Predicted Cmax (ng/mL)	Observed Cmax (ng/mL)	MFE AUC	MFE Cmax
Fluoxetine oral	20 mg	Al-Tamimi et al. (2022)	822.96	424.18	21.4	10.1	1.94	2
Fluoxetine oral	20 mg	Díaz-Tufinio et al. (2023)	822.96	502.7	21.4	14.4	1.6	1.4
Fluoxetine oral	20 mg	Najib et al. (2005)	822.96	1070.12	21.4	19.6	0.76	1
Fluoxetine oral	20 mg	Negi et al. (2021)	822.96	1132.69	21.4	23.5	0.72	0.9
Fluoxetine oral	20 mg	Ravinder et al. (2013)	822.96	623.44	21.4	10.2	1.3	2
Fluoxetine oral	20 mg	Moraes et al. (1999)	822.96	531.03	21.4	12.9	1.5	1.6
Fluoxetine oral	40 mg	Addison et al. (1998)	1695.59	1907.86	42.9	35.2	0.88	1.2
Norfluoxetine	-	Addison et al. (1998)	6340.72	6049.23	21.6	22.3	1	0.9
Fluoxetine oral	40 mg	Sutherland et al. (2001)	1695.59	2065.23	42.9	27.8	0.82	1.5
Norfluoxetine	-	Sutherland et al. (2001)	6340.72	6516.29	21.6	19.4	0.97	1.1
Fluoxetine oral	40 mg	Green et al. (2002)	1695.59	2725.1	42.9	23	0.61	1.8
Norfluoxetine	-	Green et al. (2002)	6340.72	4236.43	21.6	12.39	1.4	1.7
Fluoxetine oral	40 mg	Vlase et al. (2005)	1695.59	2373	42.9	28.8	0.71	1.4
Norfluoxetine	-	Vlase et al. (2005)	6340.72	6511.9	21.6	15.28	0.97	1.4

**FIGURE 6:**
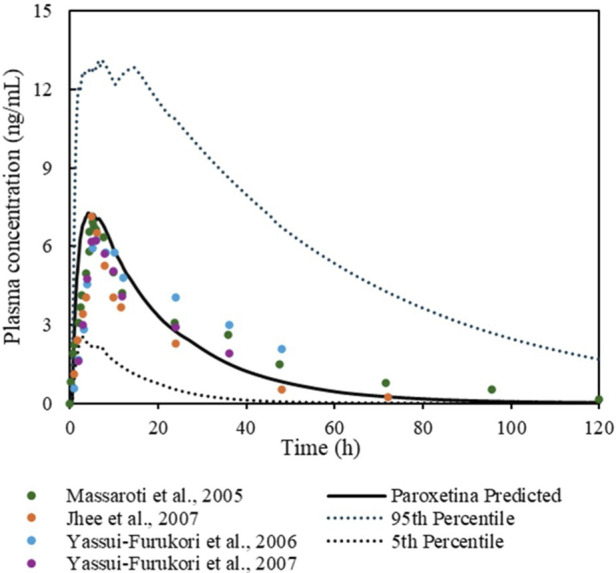
Simulation of plasma concentration–time profiles of PR.

Figure 6 shows the simulation of plasma concentration *versus* time for PR following oral administration in healthy humans. The mean AUC MFE and Cmax were 1.3 and 1.2, respectively. The calculated MFE parameter was within the recommended range (Table 4).

**TABLE 4:** Summary of clinical study data used for PR. The table reports predicted and observed data, along with MFE for the evaluated kinetic parameters. Observed AUC values were extrapolated up to 120 h.

Compound/route of administration	Dose	Ref	Predicted AUC (ng·h/mL)	Observed AUC (ng·h/mL)	Predicted Cmax (ng/mL)	Observed Cmax (ng/mL)	MFE AUC	MFE Cmax
Paroxetine oral	20 mg	Massaroti et al. (2005)	181.49	111.79	8.1	6.9	1.6	1.1
Paroxetine oral	20 mg	Jhee et al. (2011)	181.49	105.74	8.1	7.1	1.7	1.1
Paroxetine oral	20 mg	Yasui-Furukori et al. (2006)	181.49	206.59	8.1	6.2	0.87	1.3
Paroxetine oral	20 mg	Yasui-Furukori et al. (2006)	181.49	141.06	8.1	6.2	1.2	1.3

### 3.4 Ayahuasca and SSRIs: interaction simulations

**FIGURE 7:**
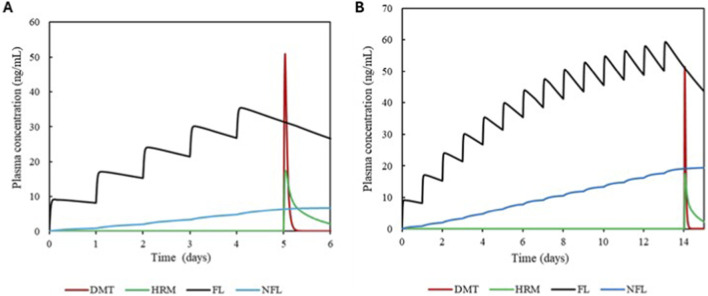
Plasma simulation of DMT and HRM in the presence of the inhibitor fluoxetine. Panel **(A)** shows the simulation over 6 days, and panel **(B)** shows the simulation over 14 days.

**FIGURE 8:**
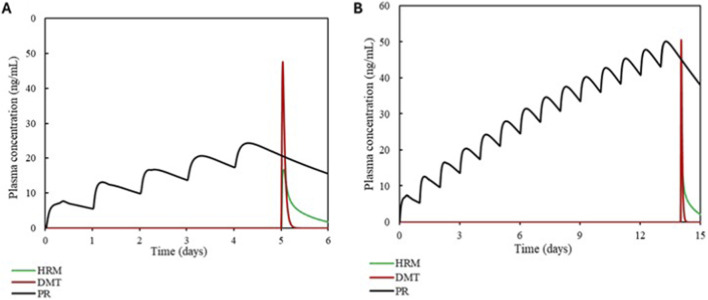
Plasma simulation of DMT and HRM in the presence of the inhibitor paroxetine. Panel **(A)** shows the simulation over 6 days, and panel **(B)** shows the simulation over 14 days.

The simulations of the interactions between the alkaloids and synthetic drugs are shown in Figures 7, 8 and were performed using FL and PR as CYP2D6 inhibitors.

The influence of DMT and HRM in a protocol simulating 6- and 14-day SSRI administration revealed the following results.

Coadministration of FL resulted in an average increase in HRM’s AUC of 1.27-fold in and an increase in Cmax of 1.15-fold. For DMT, FL coadministration resulted in AUC increase of 2.1-fold and a Cmax increase of 2.22-fold. Complete data are detailed in Table 5.

**TABLE 5:** Simulated pharmacokinetic parameters for DMT and HRM in the absence and presence of the inhibitor FL. The table presents the ratios of AUC and Cmax after 5- and 14-day simulations.

Substrate/Dose (mg/kg)	Inhibitor/Dose (mg)	Simulation duration	AUC ratio substrate	Cmax ratio substrate
DMT 0.67	Fluoxetine 20	5 days	2.2	2.2
HRM 1.77	Fluoxetine 20	5 days	1.26	1.15
DMT 0.67	Fluoxetine 20	14 days	2.1	2.24
HRM 1.77	Fluoxetine 20	14 days	1.28	1.15

In the presence of PR, HRM showed an average increase of 1.22-fold in the AUC and 1.12-fold in the Cmax. DMT exhibited a 2.13-fold increase in both AUC and Cmax. Complete data are detailed in Table 6.

**TABLE 6:** Simulated pharmacokinetic parameters for DMT and HRM in the absence and presence of the inhibitor PR. The table presents the AUC, Cmax values, and their corresponding ratios after 5- and 14-day simulations.

Substrate/Dose (mg/kg)	Inhibitor/Dose (mg)	Simulation duration	AUC ratio substrate	Cmax ratio substrate
DMT 0.67	Paroxetine 20	5 days	2.11	2
HRM 1.77	Paroxetine 20	5 days	1.20	1.11
DMT 0.67	Paroxetine 20	14 days	2.16	2.26
HRM 1.77	Paroxetine 20	14 days	1.25	1.14

### 3.5 Sensitivity analysis

The sensitivity analyses of DMT Tmax and Cmax indicated that physicochemical parameters, particularly lipophilicity (logP), exert a predominant influence on both the time to reach maximum concentration and the maximum plasma concentration, suggesting that logP-related processes play a central role in the kinetics and extent of absorption. Additionally, gastric emptying time showed a moderate influence on both parameters, indicating that gastrointestinal transit contributes to the observed delay in Tmax and to the modulation of Cmax. The results of these analyses are presented in Supplementary Figure S7.

Furthermore, sensitivity analysis of systemic exposure revealed that MAO-A–related parameters were the main determinants of DMT AUC and Cmax in the presence of paroxetine, with MAO-A–mediated clearance being the most influential factor. Parameters associated with time-dependent inhibition of CYP2D6 showed a moderate influence, whereas Ki values related to reversible CYP2D6 inhibition had minimal impact under the simulated conditions. For HRM, CYP2D6-related parameters contributed more substantially to variability in systemic exposure, reflecting the role of this enzyme in its clearance. These results are presented in Supplementary Figure S8 of the Supplementary Material.

## 4 Discussion

### 4.1 PBPK model development and verification

#### 4.1.1 HRM and DMT

The HRM and DMT PBPK models were developed with the aim of quantitatively describing the systemic exposure of both alkaloids and supporting their use in mechanistic interaction simulations. Incorporation of *in vitro* clearance data from MAO-A and cytochrome P450 pathways (Yu et al., 2003; Good et al., 2023) provided a mechanistically grounded metabolic framework. The selected distribution approaches—particularly the Rodgers and Rowland method—successfully captured the extensive tissue penetration of HRM, consistent with its high lipophilicity (LogP 3.5) and long half-life driven by redistribution from lipid-rich compartments (Rodgers and Rowland, 2007). The Tmax observed for DMT in Figure 5 occurred later than predicted by the model, which may be related to different physiological and experimental factors. Ayahuasca ingestion, as a complex plant-based matrix, may alter gastrointestinal transit, promoting gradual compound release and delayed absorption. Sensitivity analysis indicated that physicochemical parameters, particularly lipophilicity (logP), exert a greater influence on both Tmax and Cmax, suggesting that logP-related processes play a relevant role in the absorption kinetics of DMT. Additionally, gastric emptying time showed an influence on both parameters, indicating that gastrointestinal transit contributes to the observed delay in Tmax and to the modulation of Cmax, although to a lesser extent when compared to physicochemical factors. Although published CYP2D6 turnover parameters for HRM (e.g., kcat = 29.7 min^-1^) did not fully reproduce clinical exposure, model refinement through PK-Sim’s Parameter Identification tool yielded an optimized kcat that better reflected observed *in vivo* kinetics. This is consistent with other PBPK studies, where CYP2D6 parameters often require adjustment to account for genetic variability, assay system limitations, and differences in catalytic efficiency across experimental conditions (Rüdesheim et al., 2020; Taylor et al., 2020). Given the known 50–60-fold variability in CYP2D6 activity -individuals, the optimized value remains biologically and clinically plausible (Pan et al., 2011; Johansson et al., 2025).

In contrast to HRM, DMT displayed rapid clearance and limited tissue retention, consistent with its physicochemical properties and previously described PK behaviour (Cameron and Olson, 2018). The model accurately predicted the short-lived systemic exposure of DMT and reproduced the timing of maximal concentrations reported in human studies. Importantly, incorporation of an HRM–DMT inhibitory interaction (via MAO-A) allowed the PBPK model to mechanistically capture the enhanced DMT bioavailability resulting from β-carboline co-administration—a hallmark of the ayahuasca pharmacological profile.

Collectively, the successful qualification of both models confirms that they are suitable for DDI simulations involving CYP2D6 inhibitors and support the overarching objective of predicting clinically relevant exposure changes under different antidepressant treatment conditions.

#### 4.1.2 Fluoxetine and paroxetine

The PBPK models developed for FL, NFL, and PR adequately reproduced their observed pharmacokinetic behaviour, supporting their application in mechanistic interaction simulations with ayahuasca alkaloids. The Schmitt distribution model, used for FL and NFL, accounted for their marked lipophilicity and extensive tissue distribution, consistent with known accumulation in adipose tissue and the central nervous system (Schmitt, 2008).

Fluoxetine presents unique modeling challenges due to its strong inhibition of its own CYP2D6-mediated metabolism, resulting in prolonged half-life and nonlinear accumulation (Hamelin et al., 1996). Incorporating the contributions of CYP2D6, CYP2C9, and CYP3A4—supported by previous mechanistic studies allowed a more realistic representation of FL → NFL biotransformation. Parameter identification improved the alignment between simulated and observed profiles, particularly during chronic dosing, when metabolic auto-inhibition becomes more pronounced.

The PR model, aligned with the work of Rüdesheim et al. (2022), captured its high permeability, substantial first-pass metabolism, and dual dependence on CYP2D6 and CYP3A4 for clearance (Cho et al., 2024). Optimization of CYP2D6 kcat improved predictions of both peak concentrations and overall exposure. The use of the Rodgers and Rowland distribution method adequately reflected PR’s behaviour as a lipophilic base with high tissue solubility at physiological pH (Rodgers and Rowland, 2007).

Both SSRI models behaved as expected for strong CYP2D6 inhibitors, providing a robust foundation for evaluating their impact on the metabolic disposition of HRM and DMT.

### 4.2 Interaction analysis

The simulations demonstrated that both paroxetine and fluoxetine significantly alter the pharmacokinetic profiles of HRM and, consequently, of DMT. These findings are consistent with their well-established inhibitory potency toward CYP2D6, an enzyme that contributes meaningfully to HRM clearance (Yasui-Furukori et al., 2006). In particular, PR acts as a time-dependent inhibitor of CYP2D6, exhibiting cumulative inhibitory effects after repeated administration. Considering that antidepressant use typically occurs on a chronic basis, the simulated scenarios assumed prior presence of the inhibitor in the body, thereby reflecting clinically realistic steady-state conditions at the time of coadministration. As expected, increases in AUCR and CmaxR were observed during coadministration, reflecting the time-dependent nature of PR inhibition and the sustained inhibitory effect of FL and its metabolite NFL. When interpreted according to the EMA/ICH M12 framework, the magnitude of these interactions falls within clinically meaningful categories: HRM exposure increased by approximately 1.2–1.3-fold, a range classified as a weak interaction, whereas DMT exposure increased by approximately 2.1-fold, which constitutes a moderate interaction (ICH, 2025).

Although moderate DDIs are not always considered clinically prohibitive, their significance must be evaluated in light of the underlying pharmacology. DMT possesses relatively narrow tolerability margins when MAO-A inhibition is present, and even modest increases in exposure may intensify serotonergic, cardiovascular, and perceptual effects (McKenna and Riba, 2015). Human studies typically report peak DMT concentrations between 10 and 50 ng/mL (Good et al., 2023; Van Der Heijden et al., 2025), the simulated increases observed in the presence of SSRIs approach or exceed the upper boundary of this range. These elevations are clinically relevant because HRM accumulation can deepen MAO-A inhibition, further enhancing DMT’s systemic availability and amplifying the overall pharmacodynamic effect. This mechanistic interpretation is further supported by the sensitivity analysis, which identified MAO-A–related parameters as the dominant determinants of DMT exposure in complex DDI scenarios involving paroxetine. Although CYP2D6 does not directly govern DMT clearance to the same extent, its functional relevance emerges indirectly through modulation of HRM exposure, leading to enhanced MAO-A inhibition. In addition, CYP2D6 remains a key determinant of HRM pharmacokinetics, particularly under conditions of time-dependent inhibition, a scenario that is clinically plausible given the chronic use of antidepressants such as paroxetine. From a mass balance perspective, the observed effects reflect the redistribution of metabolic clearance among competing pathways. Inhibition of CYP2D6 reduces HRM clearance, leading to increased systemic exposure to HRM and enhanced inhibition of MAO-A, which represents the rate-limiting pathway for DMT clearance. This sequence of events can be interpreted as a network effect, in which modulation of a secondary metabolic pathway propagates through the enzymatic system, indirectly impacting the dominant clearance pathway. Consequently, even moderate changes in a secondary metabolic pathway may result in relevant increases in systemic exposure when they indirectly affect the dominant clearance pathway of the system. The potential clinical consequences extend beyond pharmacokinetics. SSRIs elevate synaptic serotonin by inhibiting reuptake, while DMT directly stimulates serotonin receptors, including 5-HT_2_A and 5-HT_1_A (Colosimo et al., 2024). Their combined use may therefore produce a synergistic enhancement of serotonergic signaling, increasing the risk of serotonin syndrome. This phenomenon is supported by mechanistic and preclinical evidence: irreversible MAO-A inhibition can increase tyramine sensitivity by 10–30-fold (Zimmer et al., 1990), and chronic MAO-A inhibition has been shown to elevate cortical norepinephrine release approximately fourfold (Finberg et al., 1993). These data illustrate how metabolic inhibition can significantly potentiate monoaminergic responses, producing physiological and behavioral effects far greater than predicted by PK changes alone.

In this context, the PBPK simulations provide mechanistic confirmation of a clinically relevant risk associated with the concomitant use of SSRIs and ayahuasca. The results align directly with the objectives of this study by demonstrating, in a quantitative manner, how CYP2D6 inhibition can increase systemic exposure to both HRM and DMT, thereby heightening the potential for adverse serotonergic events. Given that individuals who use ayahuasca recreationally or ritually may already be undergoing antidepressant therapy—and often without medical disclosure—these findings underscore an important public health and clinical safety concern. The PBPK framework presented here offers a valuable tool to predict such interaction risks in scenarios where controlled clinical studies are impractical or ethically unfeasible.

## 5 Conclusion

PBPK modeling proved to be an effective mechanistic approach for characterizing the pharmacokinetics of DMT and HRM following oral administration of ayahuasca and for predicting their interactions with commonly prescribed selective serotonin reuptake inhibitors. The models adequately reproduced observed human plasma concentration–time profiles and mechanistically captured the influence of CYP2D6 inhibition on HRM disposition and, indirectly, on DMT bioavailability. Simulations indicate that coadministration of potent CYP2D6 inhibitors, such as fluoxetine and paroxetine, may result in clinically relevant increases in systemic exposure to HRM and moderate increases in DMT exposure, potentially elevating the risk of serotonergic effects. These findings have important clinical implications, particularly given that individuals receiving chronic antidepressant therapy may seek ayahuasca use outside supervised clinical settings. Despite these contributions, the study is limited by the scarcity of well-controlled human pharmacokinetic data for DMT and HRM and by the complexity of fluoxetine metabolism, which constrains full mechanistic characterization. Moreover, although increased systemic DMT exposure is predicted under CYP2D6 inhibition scenarios, definitive conclusions regarding the risk of serotonin toxicity cannot be drawn due to the lack of an established PK/PD relationship between DMT brain concentrations and toxicodynamic outcomes.

## Data Availability

The original contributions presented in the study are included in the article/Supplementary Material, further inquiries can be directed to the corresponding authors.
