# Supplementary material for: Predicting drug–drug interactions between ayahuasca alkaloids and SSRIs using physiologically based pharmacokinetic modeling
Source: Front Mol Biosci. 2026 Feb 18;13:1768402. doi: 10.3389/fmolb.2026.1768402 (PMC12957174; doi:10.3389/fmolb.2026.1768402)

Supplementary Material

**S1**. Chromatograms generated after extracting the data from each transition in the MassLynx™ software. The image shows the chromatographic analysis of HRM (A), DMT-D6/internal standard (B), and DMT (C).


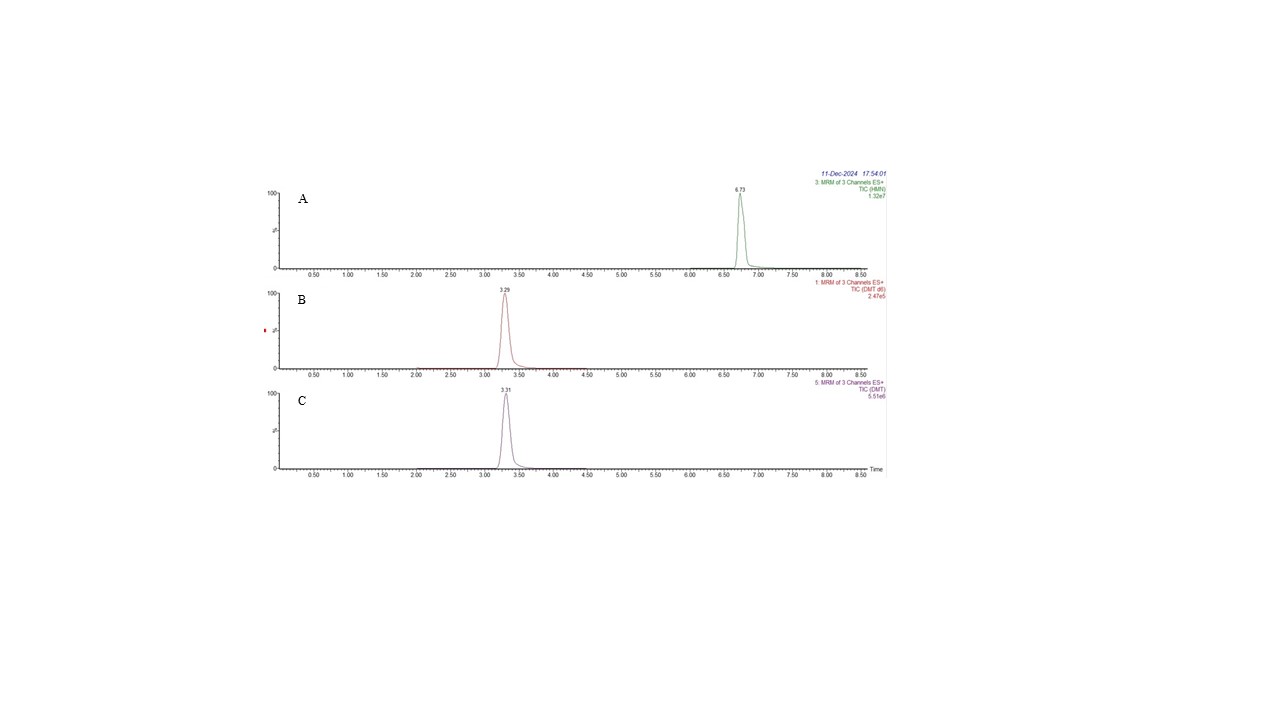


**S2.** Compiled and adjusted data for the development and refinement of the PBPK models of HRM and DMT.

| **Parameters** | | **DMT** | | | | | **HRM** | | | | | | | | | | | **Reference** | | | | | | | | | | | |
| --- | --- | --- | --- | --- | --- | --- | --- | --- | --- | --- | --- | --- | --- | --- | --- | --- | --- | --- | --- | --- | --- | --- | --- | --- | --- | --- | --- | --- | --- |
| **Physicochemical properties** |  | | | | | | | | | | | |  | | |  | | | | | | | |  |  |  |  |  |  |
| Molecular weight (g/mol) | | | 188.27 | | | | | | | | | 212.27 | | | | | | | PubChem Compound | | | | | | | | | |  |
| Log P | | | 2,75 | | | | | | | | | 3,5 | | | | | | | DrugBank | | | | | | | | | |  |
| Type of compound | | | Base | | | | | | | | | Base | | | | | | | PubChem Compound | | | | | | | | | |  |
| pKa | | | 8.6 | | | | | | | | | 8.8 | | | | | | | PubChem Compound | | | | | | | | | |  |
| Solubility | | | 1.69 mg/mL | | | | | | | | | 30.6 μg/mL | | | | | | | DrugBank | | | | | | | | | |  |
| **Blood binding properties** | | | | | | | | | | | | | | | | | | | | |  |  |  |  |  |  |  |  |  |
| Plasma protein binding | | | 32.30% | | | | | | 78.6% | | | | | | | Good et al., 2023; ADMET predictor | | | | | | | |  |  |  |  |  |  |
| **Model** | | | | Whole-body  PBPK model | | | | | | | | Whole-body  PBPK model | | | | |  | | | | | | | | |  |  |  |  |
| **Permeability** | | | 1.02 cm/min | | | | | | | | 3.96 cm/min | | | | | | | Calculated in  Pk-Sim Thelen et al., 2011 | | | | | | | | | |  |  |
| **Distribution method** | | | Rodgers  e Rowland | | | | | Rodgers  e Rowland | | | | | | | | | | Rodger e Rowland, 2007 | | | | | | | | | |  |  |
| **Clearance** | | | Enzyme kinetics | | | | | Enzyme kinetics | | | | | | | | | |  | | | | | | | | | |  |  |
| **MAO-A** Cl *in vitro*, for liver microssomes, First Order | | | 175 μl/min/mg mic. proteina | | | | | | - | | | | | Good et al., 2023 | | | | | | | | | | |  |  |  |  |  |
| **CYP2D6**  DMT (*in vitro*, for liver microssomes First Order)  HRM (presence of recombinant CYPs) | | | 29 μl/min/mg mic. protein | | | | | | | | | k_cat_ 180 1/min  (Optim.)  K_m_ 7.4 μM | | | | | | | | Good et al., 2023; Yu et al., 2003 | | | | | | | | |  |
| **CYP2C19**  **HRM** (presence of recombinant CYPs) | | | | | - | | | | | | | k_kat_ 21.4 1/min  K_m_ 121 μM | | | | | | | Yu et al., 2003 | | | | | | | |  |  |  |
| **CYP2C9** (presence of recombinant CYPs) | | | | | - | | | | | | | k_kat_ 11.9 1/min  K_m_ 117 μM | | | | | | | Yu et al., 2003 | | | | | | | | | |  |
| **CYP1A1** (presence of recombinant CYPs) | | | | | - | | | | | k_kat_ 9.23 1/min  K_m_ 52 μM | | | | | | Yu et al., 2003 | | | | | | | |  |  |  |  |  |  |
| **CYP1A2** (presence of recombinant CYPs) | | | | | | - | | | | k_kat_ 9.24 1/min  K_m_ 14.7 μM | | | | | Yu et al., 2003 | | | | | | |  |  |  |  |  |  |  |  |
| **Inhibition data** | | | | | Inhibition constant | | | | | - | | | | |  | | | | | | | |  |  |  |  |  |  |  |
| K_i_ harmine/MAO-A (μM) | | | | | - | | | | | 0.005 | | | | | Kim et al., 1997 | | | | | | |  |  |  |  |  |  |  |  |

LogP: Octanol–water partition coefficient; pKa: Acid dissociation constant; K_i_: Inhibition constant; K_m_: Michaelis–Menten constant; V_max_: Maximum rate of the enzymatic reaction; K_kat_: Catalytic constant Cl: Clearance; Otim: optimized.

**S3.** Compiled and adjusted data for the development and refinement of the PBPK models of FL, NFL and PR.

| **Parameters** | | FL | NFL | PR | | Reference | | | |  |  |
| --- | --- | --- | --- | --- | --- | --- | --- | --- | --- | --- | --- |
| **Physicochemical properties** | | | | | | | | |  |  |  |
| Molecular weight (g/mol) | | 345.8 | 295.3 | 329.4 | | DrugBank | | | |  |  |
| Log P | | 4.09 | 3.8 | 4.12 | | DrugBank | | | |  |  |
| Type of compound | | Base | - | Base | | DrugBank | | | |  |  |
| pKa | | 9.8 | 9 | 9.9 | | DrugBank | | | |  |  |
| Solubility (mg/mL) | | 103 | - | 7.31 | | DrugBank | | | |  |  |
| **Blood binding properties** | | | | | | | |  |  |  |  |
| Plasma protein binding | | 94% | 87% | 95% | | DrugBank | | | |  |  |
| Permeability | | 9.38E-4 cm/s |  | 3.9E-5 cm/min | | Calculated in Pk-Sim  Ingels et al., 2004 | | | |  |  |
| **Model** | Whole-body PBPK model | | | | | |  | | | |  |
| **Distribution method** | | Schmitt | Schmitt | Rodgers e Rowland | | Rodgers et al. 2007; Schmitt, 2004 | | | |  |  |
| **Clearance** | Enzyme kinetics | | | |  | | | | | | |
| **CYP2D6**  FL in *vitro metabolic rate in the* presence of recombinant CYPs  Micaelis Menten  NFL in *vitro metabolic rate in the* presence of recombinant CYPs First order  PR- *in vitro* Cl Michaelis Menten | 0.86 pmol/min/pmol rec enzyme  K_m_: 0.23  (Optim.) | | 0,98  µL/min/pmol rec. enzyme | K_m_ 0.40 µmol/L  k_cat_ 1.37 1/min | | Jeong et al., 2021; Chang-Keun et al., 2024 | | | |  |  |
| **CYP3A4**  FL – in *vitro metabolic rate in the* presence of recombinant CYPs  Micaelis Menten  PR *in vitro* Cl Michaelis Menten | 5 pmol/min/pmol rec enzyme  K_m_: 26.27  (Optim.) | | - | K_m_ 4.32 µmol/L  k_cat_ 1.01 1/min | | Jeong et al., 2021; Chang-Keun et al., 2024 | | | |  |  |
| **CYP2C19**  FL in *vitro metabolic rate in the* presence of recombinant CYPs  Micaelis Menten | 8.82 pmol/min/pmol rec enzyme  K_m_: 22.77  (Optim.) | | - | - | | Jeong et al., 2021 | | | |  |  |
| **Inespecif clearance** | 0.80 mL/min/kg | |  |  | |  | | | |  |  |
| **Renal Cl** L/h/kg | 0.30 mL/min/kg | | 0.17 | GFR fration 1 | | Jeong et al., 2021; Chang-Keun et al., 2024 | | | |  |  |
| **Inhibition data** |  | |  |  | |  | | | |  |  |
| K_i_ CYP2D6 | 0.2 µM | | 0.01 µM | K_inact_ 0.17 1/min  K_i_ 0.09 µmol/L | | Jeong et al., 2021; Chang-  Keun et al., 2024 | | | |  |  |
| K_i_ CYP3A4 | 83 µM | | 11 µM | 4.48 µmol/L | | Jeong et al., 2021; Chang-k  Keun et al., 2024 | | | |  |  |
| K_i_ CYP2C19 | 5.2 µM | | - | - | | Jeong et al., 2021 | | | |  |  |

LogP: Coeficiente de partição octanol-água; pKa: Acid dissociation constant; K_i_: Inhibition constant; K_m_: Michaelis–Menten constant; V_max_: Maximum rate of the enzymatic reaction;K_i_: Catalytic constant Cl: Clearance; GFR: Glomerular Filtration Rate.

**S4.** Demographic data of the volunteers from the conducted clinical study used for the validation of the PBPK models of DMT and HRM.

| Volunteer | Age (years) | Sex | Weight (kg) | Height (m) | Administered dose of DMT (mg/kg) | Administered dose of HRM (mg/kg) |
| --- | --- | --- | --- | --- | --- | --- |
| 1 | 29 | M | 74 | 1.78 | 0.67 | 1.77 |
| 2 | 25 | M | 79 | 1.75 |  |  |
| 3 | 24 | F | 57 | 1.59 |  |  |
| 4 | 26 | M | 75 | 1.73 |  |  |
| 5 | 56 | F | 78 | 1.68 |  |  |
| 6 | 34 | M | 87 | 1.75 |  |  |

M: Women; F: Female

**S5.** Summary of clinical study data on the pharmacokinetic disposition of FL.

| Compound/Route of administration | Dose (mg) | | Population | Mean weigh (kg) | | Dosing regimen | | N | Average age (years) | Study duration (h) | | | | Female (%) | | Ref. |  |
| --- | --- | --- | --- | --- | --- | --- | --- | --- | --- | --- | --- | --- | --- | --- | --- | --- | --- |
| Fluoxetine | 20 | Healthy | | - | Sigle dose | | 46 | | 18-50 | | 150 | | 0 | | 1 | | |
| Fluoxetine | 20 | Healthy | | - | Sigle dose | | 24 | | 10-50 | | | 70 | 20 | | 2 | | |
| Fluoxetine | 20 | Healthy | | 71-94 | Sigle dose | | 24 | | 19-32 | | | 400 | 0 | | 3 | | |
| Fluoxetine | 20 | Healthy | | - | Sigle dose | | - | | - | | | 80 | 0 | | 4 | | |
| Fluoxetine | 20 | Healthy | | - | Sigle dose | | 6 | | - | | | 75 | - | | 5 | | |
| Fluoxetine | 20 | Healthy | | 49-80 | Sigle dose | | 24 | | 18-43 | | | 700 | 50 | | 6 | | |
| Fluoxetine | 40 | Healthy | | - | Sigle dose | | 26 | | - | | | 600 | - | | 7 | | |
| Fluoxetine | 40 | Healthy | | - | Sigle dose | | 24 | | - | | | 500 | - | | 8 | | |
| Fluoxetine | 40 | Healthy | | - | Sigle dose | | 26 | | - | | | 800 | 0 | | 9 | | |
| Fluoxetine | 40 | Healthy | | - | Sigle dose | | 1 | | - | | | 800 | 0 | | 10 | | |

Al-Tamini et al., 2022^1^; Díaz-Tufinio et al., 2023^2^; Najib et al., 2005^3^; Negi et al., 2021^4^; Ravinder et al., 2013^5^; Moraes et al., 1999^6^; Addison et al., 1998^7^; Sutherland et al., 2001^8^; Green et al., 2002^9^; Vlase et al., 2005^10^.

The symbol ‘–’ indicates information not provided in the study.

**S6**. Summary of clinical study data on the pharmacokinetic disposition of PR.

| Compound/Route of administration | Dose (mg) | Population | | | Mean body weigh (kg) | Dosing regimen | N | Average age (years) | Study duration (h) | Female (%) | Ref. |  |  |
| --- | --- | --- | --- | --- | --- | --- | --- | --- | --- | --- | --- | --- | --- |
| Paroxetine | 20 | | Healthy | - | | Single dose | 28 | 18-50 | 120 | 0 | 1 |  |  |
| Paroxetine | 20 | | Healthy | 69-81 | | Single dose | 24 | 19-27 | 80 | 0 | 2 | |  |
| Paroxetine | 20 | | Healthy | 46-75 | | Single dose | 12 | 20-35 | 48 | 25 | 3 | |  |
| Paroxetine | 20 | | Healthy | 45-67 | | Single dose | 13 | 21-35 | 36 | 23 | 4 | | |

Massaroti et al., 2005^1^; Jhee et al., 2007^2^; Yassui-Furukori et al., 2006^3^; Yassui-Furukori et al., 2007^4^

The symbol ‘–’ indicates information not provided in the study.

**S7.** Sensitivity analysis of DMT pharmacokinetic parameters. (A) Sensitivity of DMT Cmax and (B) Tmax

**
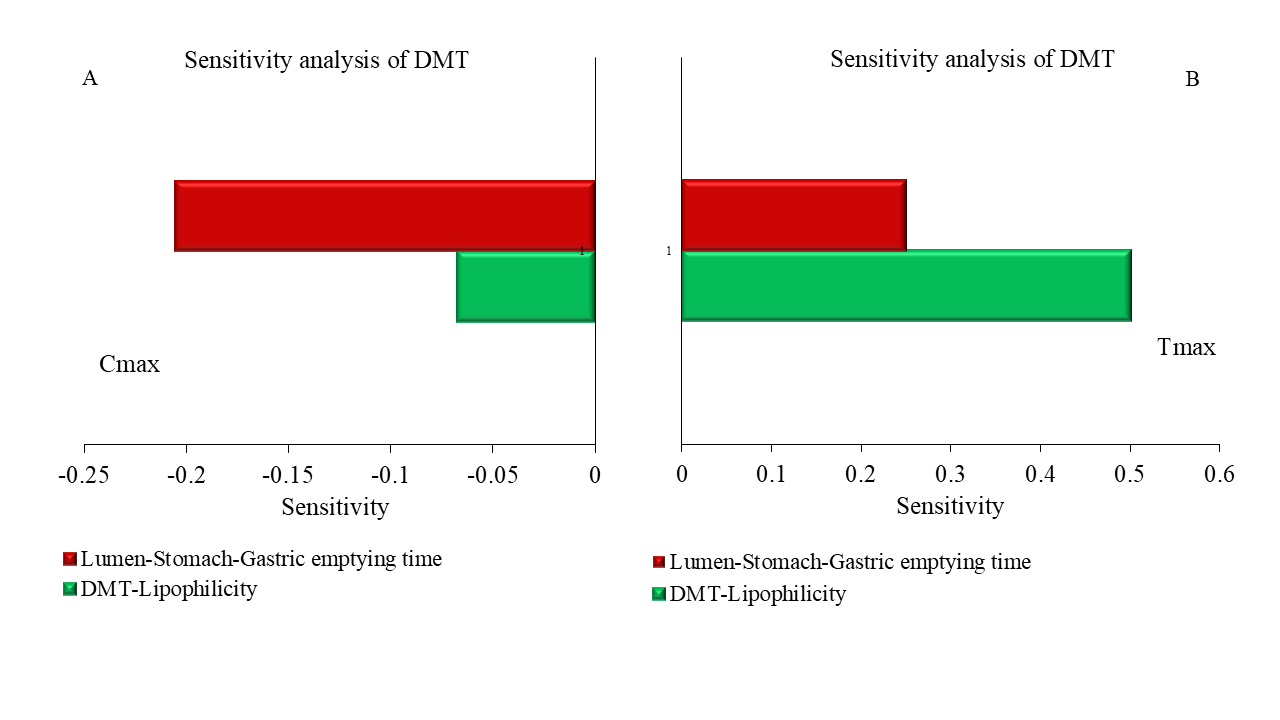
**

**S8.** Sensitivity analysis of metabolic parameters involved in complex drug–drug interaction scenarios. (A) Sensitivity of AUC and (B) sensitivity of Cmax for DMT and HRM in response to variations in parameters associated with MAO-A– and CYP2D6-mediated clearance under conditions of clinical CYP2D6 inhibition.


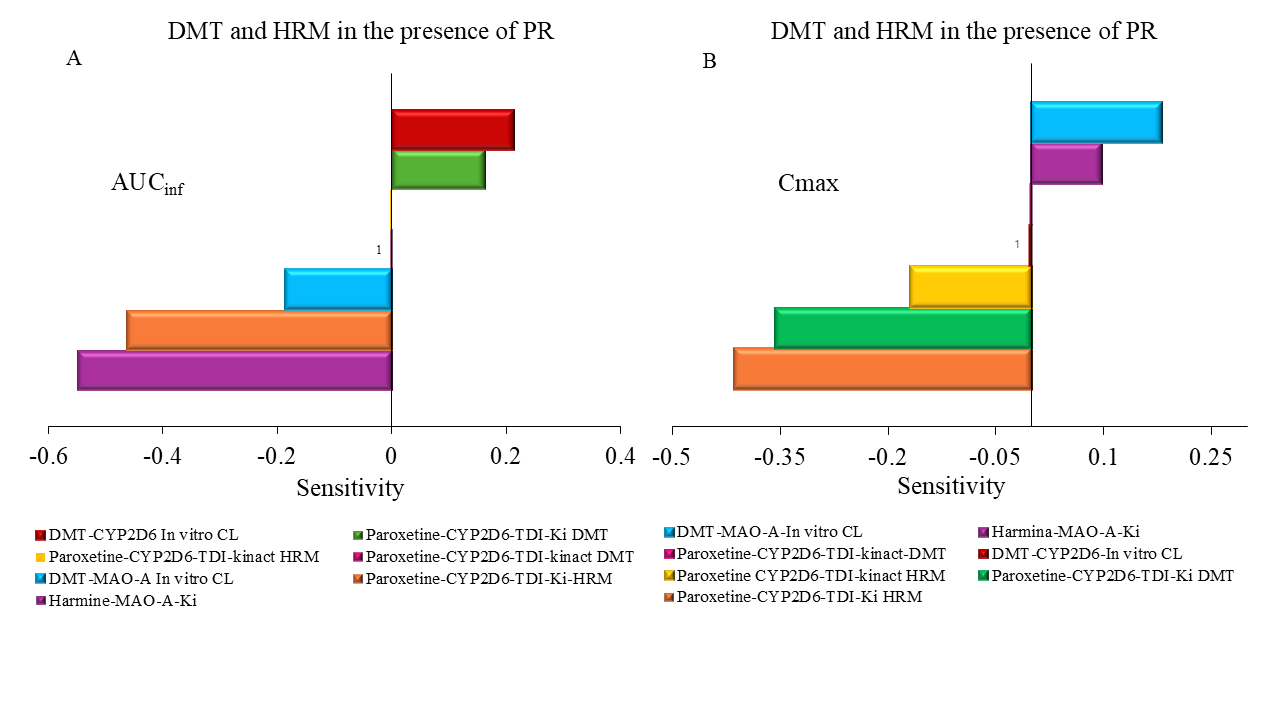

Supplement: Supplementary file 1 [file Supplementaryfile1.docx]
